# Unraveling Hypocellular AML: Insights into Diagnosis and Management

**DOI:** 10.51894/001c.144552

**Published:** 2025-09-30

**Authors:** Zijin Lin

**Affiliations:** 1 College of Osteopathic Medicine Michigan State University

## 98

### INTRODUCTION

Acute myeloid leukemia (AML) is a hematologic malignancy driven by somatic mutations that impair differentiation and promote clonal proliferation of myeloid precursors. The RUNX1 mutation, found in ~15% of adult de novo AML cases—particularly older males—is linked to treatment resistance and poor prognosis. While AML typically presents with hypercellular bone marrow, hypocellular AML is a rarer subset with <20% cellularity at diagnosis, often mimicking marrow failure syndromes like myelodysplastic syndrome (MDS). This case highlights a patient with hypocellular AML associated with a rare RUNX1 mutation, underscoring diagnostic and management challenges.

### CASE PRESENTATION

A 65-year-old male with rheumatoid arthritis developed worsening pancytopenia after methotrexate exposure. He received leucovorin rescue, and peripheral blood smear showed 3% blasts. Bone marrow biopsy revealed 10% cellularity with 50% blasts, consistent with AML. Next-generation sequencing (NGS) identified a RUNX1 mutation. Interestingly, his blood counts improved without treatment, and a repeat biopsy showed 5% blasts.

After six months of observation, he deteriorated, requiring hospitalization for neutropenic fevers. A repeat biopsy confirmed hypocellular AML with 22% blasts and 15% cellularity; the RUNX1 mutation persisted with a variant allele frequency (VAF) of 14.6%. Given his persistent pancytopenia, a multidisciplinary team initiated azacitidine and venetoclax, leading to remission by day 28. He was later referred for bone marrow transplantation.

### DISCUSSION

Repeated biopsies confirmed hypocellularity and a RUNX1 mutation, presenting a mixed picture of hypocellular AML and MDS. RUNX1 mutations are associated with MDS progression to AML, and hypocellularity is frequently seen in treatment-related MDS/AML. Methotrexate exposure may have disrupted immunosurveillance, enabling clonal expansion.

Despite standard AML treatment involving anthracycline and cytarabine, the patient’s frailty and disease features warranted azacitidine and venetoclax. His case highlights the need for personalized treatment strategies and further research into the molecular and clinical behavior of hypocellular AML.

